# Supplementary material for: Confidence interval forecasting model of small watershed flood based on compound recurrent neural networks and Bayesian
Source: PLoS One. 2025 Apr 21;20(4):e0321583. doi: 10.1371/journal.pone.0321583 (PMC12011224; doi:10.1371/journal.pone.0321583)
Supplement: Attachment — Submitted filename: Response-to-Reviewers-1231.docx [file pone.0321583.s002.docx]

**Original Article Title: “Confidence interval forecasting model of small watershed flood based on compound Recurrent Neural Networks and Bayesian**” (PONE-D-24-42128)

**To:** PLOS ONE Editor

**Re:** Response to reviewers

Dear reviewers and editors,

Thank you for allowing a resubmission of our manuscript, with an opportunity to address the reviewers’ comments.

We are uploading (a) our point-by-point response to the comments (below) (response to reviewers), (b) an updated manuscript with red highlighting indicating changes (*Supplementary Material for Review*), and (c) a clean updated manuscript without highlights (*Main Manuscript).*

Best regards,

Songsong Wang, Ouguan Xu

**Reviewer#1, Concern # 1:** Lack of comprehensive comparison experiments: The authors only compared the performance of three different models within the RNN+Bayesian framework. Methods such as ensemble approaches and attention-based models are also commonly used in time-series forecasting tasks. Including these comparison experiments would more strongly demonstrate the practical value of the proposed model.

**Author response:** Thank you for pointing this out. The reviewer is correct, and we have revised it.

**Author action:** We updated the relevant contents in the paper as follows.

We have employed various integrated regression of Machine Learning (ML) in conjunction with Bayesian methods to ascertain the optimal combination for forecasting.

We use Random Forest Regression (RFR) and Bayesian combination. RFR is an improvement over bagged decision tree. The decision tress that handles continuous variables are known as regression trees. Regression trees work similarly as decision trees by splitting each node based on a node impurity measure. We use the Sklearn Library (https://scikit-learn.org/), call the Application Programming Interface (API) of ML model directly. Through automatic parameter tuning, we obtained parameter (random_state = 60). RFR belongs the regressive prediction, the circular part of RNNs cannot be used, and the information on time series cannot be obtained. The deterministic prediction part of RNNs is weak, and its effect becomes worse after superposition of Bayesian.

For the current general-purpose attention-based models, we have tested the Transformer combined with Bayesian, and have not achieved good results. Of course, this does not mean that the transformer model is not suitable for prediction. The main reason is that it requires a lot of calculation and parameter adjustment. At present, it is less applied to time series prediction. The regression method of non RNN model cannot replace the deterministic prediction method of RNN series, which is the reason why RNN series is widely used in time series forecasting.

**Reviewer#1, Concern # 2:** Lack of ablation experiments: Ablation experiments would make the authors' arguments more well-rounded. For instance, comparing RNN+LSTM with using only LSTM could help illustrate the difference and thereby justify the use of Bayesian estimation.

**Author response:** Thank you for pointing this out. The reviewer is correct, and we have revised it.

**Author action:** We updated the relevant contents in the paper as follows.

The combination of RNNs and Bayesian improves the reliability of flood water level prediction, and makes the scheduling of flood disasters reasonably evaluated.

Although the combination of the two fully reflects the reliability and accuracy, we need to consider the role of RNN series prediction methods, and even consider whether Bayesian reduces or improves the accuracy of prediction. We use the same data as the test part in this paper, and only use the RNN series part.

Compared with the renderings of the experiment results in this paper, the accuracy of RNN alone is not as good as that combined with Bayesian. We calculated that Bayesian improved the accuracy of RNN series by about 15% on average, mainly because Bayesian method involves many RNN training to explore the optimal distribution of prediction probability and improve the accuracy of prediction. At the same time, it also shows that Bayesian method is effective. However, the training consumes a lot of time. Generally, the training of RNN series is less than 10 minutes, which is consistent with the literature [37]. However, our fastest GRU-Bayesian also takes 43 minutes.

At the same time, the deterministic prediction RNNs can only get the definite value, cannot display the confidence interval, and the reliability of the prediction cannot be measured. Bayesian has the advantage in this respect. It is worth noting that since the Bayesian method lacks continuous prediction on the sequence between, the RNNs part of the algorithm fills this deficiency. It should be said that RNN and Bayesian interact and are indispensable.

**Reviewer#1, Concern # 3:** Some parts lack clarity: It would be best for the authors to clearly and prominently list all contributions of this paper in one section. Additionally, it should be explicitly stated that the model in the paper predicts n steps ahead based on the previous m steps. Furthermore, the authors could enhance the paper by discussing the model's performance on data with varying sequence lengths.

**Author response:** Thank you for pointing this out. The reviewer is correct, and we have revised it.

**Author action:** We updated the relevant contents in the part of Introduction.

Through an examination of the uncertainty theory pertaining to forecasting and testing, it has been determined that the forecasting capabilities of the proposed model remain within the bounds of the reliability forecasting interval, even during periods characterized by rapid fluctuations in water levels. This model achieves a commendable equilibrium between reliability and accuracy. The approach employed involves the utilization of a multidimensional disaster data input unit for water level forecasting, with LSTM networks identified as the most effective method for confidence interval forecasting. Additionally, we have employed various integrated regression of Machine Learning (ML) in conjunction with Bayesian methods to ascertain the optimal combination for forecasting. A significant contribution of this research lies in the identification of a methodology that effectively merges time series accuracy forecasting with probabilistic assessments within the context of small watershed flood forecasting. By integrating RNNs with Bayesian principles, we have developed a confidence interval forecasting model that enhances both the reliability and accuracy of flood forecasting.

We use the Time window predicts next step on the previous 120 steps. About time window, the data of flood water level at the future time point (each time is the next hour) is predicted through the data of disaster causing factors in the moving time window.

Our experimental comparisons indicate that an increase in the volume of training data does not necessarily correlate with improved prediction accuracy. As illustrated in Table 8, the MDAD of various models is minimized when the time window is set to five days. Both excessively short and excessively long time windows are detrimental to time series prediction accuracy.

**Table 8 The relationship between MDAD of prediction and the length of time window**

| **Number of days** | **1** | **3** | **5** | **7** | **10** |
| --- | --- | --- | --- | --- | --- |
| Base RNN-Bayesian | 2.02 | 1.71 | 1.22 | 1.67 | 1.91 |
| LSTM-Bayesian | 0.1 | 0.05 | 0.03 | 0.05 | 0.08 |
| GRU-Bayesian | 0.81 | 0.24 | 0.13 | 0.15 | 0.21 |
| RFR-Bayesian | 5.13 | 3.15 | 2.24 | 2.56 | 2.71 |

**Reviewer#1, Concern # 4:** The authors should clarify the meaning of the x-axis labels in the figures.

**Author response:** Thank you for pointing this out. The reviewer is correct, and we have revised it.

**Author action:** We updated the relevant figures in the paper.
