# Supplementary material for: Confidence interval forecasting model of small watershed flood based on compound recurrent neural networks and Bayesian
Source: PLoS One. 2025 Apr 21;20(4):e0321583. doi: 10.1371/journal.pone.0321583 (PMC12011224; doi:10.1371/journal.pone.0321583)
Supplement: Attachment — Submitted filename: Response-to-Reviewers-0110.docx [file pone.0321583.s003.docx]

**Original Article Title: “Confidence interval forecasting model of small watershed flood based on compound Recurrent Neural Networks and Bayesian**” (PONE-D-24-42128R1)

**To:** PLOS ONE Editor

**Re:** Response to reviewers

Dear reviewers and editors,

Thank you for allowing a resubmission of our manuscript, with an opportunity to address the reviewers’ comments.

We are uploading (a) our point-by-point response to the comments (below) (response to reviewers), (b) an updated manuscript with red highlighting indicating changes (*Supplementary Material for Review*), and (c) a clean updated manuscript without highlights (*Main Manuscript).*

Best regards,

Songsong Wang, Ouguan Xu

**Reviewer#2, Concern # 1:** Abstract: The abstract seems insufficient and lacks clarity. It does not explain the significance and practical application of the models, the input variables, the length of the statistical period, and the time scale. Adding one or two sentences could resolve these ambiguities.

**Author response:** Thank you for pointing this out. The reviewer is correct, and we have revised it.

**Author action:** We updated the relevant contents in the paper as follows.

We used the multidimensional disaster data input unit for water level forecasting, including hydrology, meteorology, and geography, and 5 days of time windows for forecasting, The comprehensive reliability of LSTM-Bayesian for 0~102 hours flood reached 92.31%, and the comprehensive accuracy reached 89.15%, and confidence interval forecasting using LSTM is the best method, and achieved reasonable balance of reliability and accuracy. Overall, compound RNN could be a good alternative for forecasting hourly streamflow and extreme water level in small watersheds.

**Reviewer#2, Concern # 2:** Structure: Unfortunately, the article does not follow the IMRAD (Introduction, Methods, Results, and Discussion) structure. Overall sectioning and subsections would help clarify the topic and improve understandability.

**Author response:** Thank you for pointing this out. The reviewer is correct, and we have revised it.

**Author action:** We updated the relevant contents in the paper as follows.

(1) We have revised the "Methods" section, "3.2 RNNs Bayesian model", to make it clearer.

(2) (2) We merged data and results to generate the "4 Data and results" chapter.

(3) Overall sectioning and subsections would help clarify the topic and improve understandability.

**Reviewer#2, Concern # 3:** Study Area Map: A map of the study area (also DEM, Land use/Land Cover maps) is not provided, nor is there sufficient explanation regarding the characteristics of the study area.

**Author response:** Thank you for pointing this out. The reviewer is correct, and we have revised it.

**Author action:** We updated the relevant contents in the paper as follows.

(1) We acknowledge that creating high-quality maps does help enhance the visualization of articles. However, in the actual operation process, we have encountered some technical and resource challenges: lack of professional mapping tools and technical capabilities, team members do not have the operational skills of advanced GIS software, and it is difficult to produce maps that meet the publishing standards in the short term. Considering the time arrangement and budget constraints of the project, it is difficult to obtain and process the required high-resolution remote sensing images and terrain data. In order to make up for the lack of providing maps directly, we added geographical introduction to help readers better understand the research background and results.

(2) We added a full explanation of the characteristics of the study area：The upper reaches of the Qiantang River are characterized by mountainous terrain, with the Qixi Reservoir monitoring point located in Kaihua County, Zhejiang Province, China, which marks the source of the Qiantang River. Kaihua County experiences a subtropical monsoon climate, situated on the northern edge of this climatic zone. This region is known for its warmth, humidity, abundant rainfall, and distinct four seasons. The annual average precipitation in Kaihua County is 1990 mm, ranking second highest in Zhejiang Province.

Rainfall in the Qiantang River watershed is primarily concentrated during the summer months (June to September), particularly during the plum rain and typhoon seasons. Heavy rainfall during these periods is the primary cause of floods in the region. The upper reaches of the Qiantang River feature steep terrain, narrow river valleys, and rapid water flow, conditions that facilitate the rapid formation of floods. Floods often develop within a short time frame, typically hours or even minutes after heavy rainfall, posing significant challenges for early warning systems and emergency response efforts.

**Reviewer#2, Concern # 4:** Statistical Characteristics: It would have been better to specify the statistical characteristics of the data used in a table.

**Author response:** Thank you for pointing this out. The reviewer is correct, and we have revised it.

**Author action:** We updated the relevant contents in the paper as follows.

We added the accuracy and reliability features, and explained and discussed them.

**Reviewer#2, Concern # 5:** Variable Combinations: The reasons for selecting combinations of input model variables should be explained, and the scenarios used should be specified in a table, including the combinations of input parameters.

**Author response:** Thank you for pointing this out. The reviewer is correct, and we have revised it.

**Author action:** We updated the relevant contents in the paper as follows. We detailed description of the problem.

**Reviewer#2, Concern # 6:** Correlation Matrix: The correlation matrix between input and output variables is not provided.

**Author response:** Thank you for pointing this out. The reviewer is correct, and we have revised it.

**Author action:** We updated the relevant contents in the paper as follows.

There are many disaster-causing factors affecting future water level prediction, including hydrology, meteorology and geography, etc. In order to obtain the factors with great influence, we use the relationship matrix, add the relationship matrix to the analysis of the relationship between the flood disaster-causing factors and the water level, as shown in Figure 4.

The future water level to be predicted is mainly related to the historical water level, up to 58%, and the relationship with rainfall is up to 28%, which is consistent with the basic law of hydrology. In addition, humidity, weather status, geography, etc. are also closely related to the future water level, and these data will be used as the input elements of the uncertainty prediction model.

**Reviewer#2, Concern # 7:** Evaluation Criteria: The evaluation criteria for the models used have not been presented. It would have been better to provide these criteria values for each model in a table to compare the results.

**Author response:** Thank you for pointing this out. The reviewer is correct, and we have revised it.

**Author action:** We updated the relevant contents in the paper as follows. e detailed description of the problem.

In the key phase of prediction, LSTM-Bayesian still maintained the advantages of the test phase, especially in the 0-72h phase, the PICP reached 96.18%, which was the closest to the actual water level, and the MDAD was only 0.01m, which had a good prediction effect, taking into account the reliability and accuracy. GRU-Bayesian also performed well, the only disadvantage is that MDAD still exists large error, and 10 times less than LSTM-Bayesian, but it is also within the acceptable range. The deviation of base RNN-Bayesian is the weakest among the three. Another obvious feature is that when the water level rises rapidly, the prediction effect is better, and the main disaster causing factors, mainly rainfall, are considered in the model. In the 72-102h phase, LSTM-Bayesian and GRU-Bayesian had a large deviation, and PICP could not cover the actual water level. base RNN-Bayesian covered the actual water level due to PICP with a large coverage rate, and the MDAD value was relatively stable. At the same time, the applicability of water level prediction was reduced due to too large PICP. In the prediction phase, 0-72h maintained the advantage, and the three predictions were effective.

To facilitate statistics and analysis, we convert PICP and CIAW into comprehensive reliability and accuracy. The reliability of base RNN-Bayesian and LSTM-Bayesian models is higher than GRU-Bayesian model, reaching 84.34% and 92.31%, respectively. The accuracy of LSTM Bayesian and GRU-Bayesian prediction models is higher than base RNN-Bayesian model, reaching 89.15% and 93.05%, respectively. Taken together, LSTM-Bayesian model has advantages in reliability and accuracy.

**Reviewer#2, Concern # 8:** Conclusion Section: In the general conclusion section, it would have been better to mention the strengths and weaknesses of the model, as well as its advantages and disadvantages.

**Author response:** Thank you for pointing this out. The reviewer is correct, and we have revised it.

**Author action:** We updated the relevant contents in the paper as follows.

The biggest contribution of the confidence interval forecasting model of small watered flood based on compound RNN and Bayesian proposed in this paper is that the RNNs-Bayesian models can consider both reliability and accuracy of flood water level prediction, which is also of great concern in hydrological forecasting. Of course, it should also be considered that the model relies on the high-quality near time series data of the monitoring points' prediction time points, and has excellent prediction effect for the short term (0~72h), but the reliability and accuracy of the medium and long-term prediction are not enough. The uncertain prediction model will play an important role in the prediction, early warning, preview and pre planning of small watershed.

**Reviewer#2, Concern # 9:** Overall Suitability: In summary, I find the article suitable for publication after making these revisions.

**Author response:** Thank you for pointing this out. The reviewer is correct, and we have revised it.

**Author action:** We have updated the relevant contents in the paper.

**Reviewer#3, Concern # 1:** Write the abstract more clear with some output values.

**Author response:** Thank you for pointing this out. The reviewer is correct, and we have revised it.

**Author action:** We updated the relevant contents in the paper as follows.

We used the multidimensional disaster data input unit for water level forecasting, including hydrology, meteorology, and geography, and 5 days of time windows for forecasting, The comprehensive reliability of LSTM-Bayesian for 0~102 hours flood reached 92.31%, and the comprehensive accuracy reached 89.15%, and confidence interval forecasting using LSTM is the best method, and achieved reasonable balance of reliability and accuracy. Overall, compound RNN could be a good alternative for forecasting hourly streamflow and extreme water level in small watersheds.

**Reviewer#3, Concern # 2:** Contribution is not clear.

**Author response:** Thank you for pointing this out. The reviewer is correct, and we have revised it.

**Author action:** We updated the relevant contents in the paper as follows.

The biggest contribution of the confidence interval forecasting model of small watered flood based on compound RNN and Bayesian proposed in this paper is that the RNNs-Bayesian models can consider both reliability and accuracy of flood water level prediction, which is also of great concern in hydrological forecasting. Of course, it should also be considered that the model relies on the high-quality near time series data of the monitoring points' prediction time points, and has excellent prediction effect for the short term (0~72h), but the reliability and accuracy of the medium and long-term prediction are not enough. The uncertain prediction model will play an important role in the prediction, early warning, preview and pre planning of small watershed.

**Reviewer#3, Concern # 3:** In the introduction and conclusion, emphasize the practical applications and real world impact of the proposed model to align the technical work with practical significance.

**Author response:** Thank you for pointing this out. The reviewer is correct, and we have revised it.

**Author action:** We updated the relevant contents in the paper as follows.

The error rate of flood warning is high, it is of great significance to study the prediction of flood in small watersheds to reduce the complex sudden flood disasters.

It improves the reliability of flood warning in small watersheds, and can provide reliable information for the decision-making of flood control and disaster management of government departments to avoid flood disasters.

**Reviewer#3, Concern # 4:** Analyse the performance of the algorithm as the prediction steps increases.

**Author response:** Thank you for pointing this out. The reviewer is correct, and we have revised it.

**Author action:** We updated the relevant contents in the paper as follows.

As the prediction steps increases, according to Figure 6, 7 and 8, the prediction confidence interval is getting larger and larger, but the ability to cover the real value is getting lower and lower, both reliability and accuracy tend to decline, a phenomenon that aligns with established principles regarding uncertainty. The reliability is not guaranteed, and the accuracy is getting lower and lower. In addition, according to Table 8, when the prediction window is the 5 days, the MDAD is the smallest. The bigger it is, the worse the comprehensive performance of reliability and accuracy is.
